# Joint motion quality in vibroacoustic signal analysis for patients with patellofemoral joint disorders

**DOI:** 10.1186/1471-2474-15-426

**Published:** 2014-12-12

**Authors:** Dawid Bączkowicz, Edyta Majorczyk

**Affiliations:** Institute of Physiotherapy, Faculty of Physical Education and Physiotherapy, Opole University of Technology, Prószkowska Street 76, PL-45-758 Opole, Poland; Laboratory of Immunogenetics and Tissue Immunology, Ludwik Hirszfeld Institute of Immunology and Experimental Therapy, Polish Academy of Sciences, Weigla Street 12, 53-114 Wrocław, Poland

**Keywords:** Vibroacoustic signal, Patellofemoral joint, Lateral patellar compression syndrome, Chondromalacia, Osteoarthritis, Quality of motion

## Abstract

**Background:**

Chondromalacia, lateral patellar compression syndrome and osteoarthritis are common patellofemoral joint disorders leading to functional and/or structural disturbances in articular surfaces. The objective of the study was to evaluate their impact on joint motion quality via the vibroacoustic signal generated during joint movement analysis.

**Methods:**

Seventy-three patients (30 with chondromalacia, 21 with lateral patellar compression syndrome, and 22 with osteoarthritis) and 32 healthy controls were tested during flexion/extension knee motion for vibroacoustic signals using an acceleration sensor. Estimated parameters: variation of mean square (VMS), difference between mean of four maximum and mean of four minimum values (R4), power spectral density for frequency of 50–250 Hz (P1) and 250–450 Hz (P2) were analyzed.

**Results:**

Vibroacoustic signals recorded for particular disorders were characterized by significantly higher values of parameters in comparison to the control group. Moreover, differences were found among the various types of patellofemoral joint disturbances. Chondromalacia and osteoarthritis groups showed differences in all parameters examined. In addition, osteoarthritis patients exhibited differences in VMS, P1 and P2 values in comparison to lateral patellar compression syndrome patients. However, only the value of R4 was found to differ between knees with lateral patellar compression syndrome and those with chondromalacia.

**Conclusion:**

Our results suggest that particular disorders are characterized by specific vibroacoustic patterns of waveforms as well as values of analyzed parameters.

**Electronic supplementary material:**

The online version of this article (doi:10.1186/1471-2474-15-426) contains supplementary material, which is available to authorized users.

## Background

The knee joint complex is the largest articulation in the human body, with each component playing a specific function. The crucial role of medial and lateral knee compartments is to transfer body weight from the femur to the tibia as well as to provide ergonomic functionality of the lower leg through flexion. The patellofemoral joint (PFJ) allows patellar movement and provides physiological function of the knee extensor mechanism. The patella, being the largest sesamoid bone, centralizes the divergent forces generated by particular heads of the quadriceps muscle[1]. Moreover, the presence of the patella improves the efficiency of the knee extension mechanism by expanding the quadriceps lever arm[2].

A physiological adaptation of the PFJ to large weight bearing is the thickest hyaline cartilage (6–7 mm) in the human body[3]. However, considerable involvement of the knee extension mechanism in daily activity causes significant generation of forces, often leading to mechanical disorders of the PFJ. In consequence, grinding of articular surfaces and disability of cartilage function are observed. Among the most common disorders of PFJ leading to anterior knee pain are lateral patellar compression syndrome (LPCS), chondromalacia (CMP) and osteoarthritis (OA)[4–6].

A basic diagnostic tool for imaging PFJ disturbances in anterior knee pain is classical radiography[7]. In advanced degeneration of the PFJ, X-ray examination correlates with arthroscopy evaluation which is used as a “gold standard”. However, the lower sensitivity and specificity of X-ray is a limitation for diagnosis of early stages of chondral disorders[8, 9]. On the other hand, availability of modern imaging methods such as magnetic resonance imaging is limited due to high expense[10, 11]. The discussed disorders may be characterized by impairment of patella motion quality and restriction as well as non-physiological tracking of its movement, usually associated with crepitus. Due to such abnormalities as well as diagnostic tool limitations, PFJ diagnostic management still includes a medical interview, observation and physical examination with pain assessment and evaluation of patellar movement range, which is measured quantitatively. In addition, it is postulated that evaluation of joint motion quality focused on movement smoothness and crepitus onset is a helpful tool in physical examination. However, such a qualitative assessment is more difficult to interpret and document due to the highly subjective nature of the examination[12]. Therefore, there have been calls for the development of more sensitive and objective methods to evaluate quality of joint motion[13–15].

Vibroarthrography (VAG) is a physical examination supporting accurate assessment of joint motion quality[16]. It is based on recording the vibroacoustic signal during movement of articular surfaces. It has been demonstrated that VAG signals generated by degenerative cartilage differ from healthy joint signals[17, 18]. Although the VAG method is still in development, it shows repeatability in subsequent motion cycles[15, 19] and in addition accuracy of over 90% when compared with the clinical findings made in the standard diagnostic procedures[11, 20].

Hence, the aim of this study is to evaluate disorder-related differences in PFJ joint motion quality using the VAG method. It may aid our understanding of the relationship between different types of articular pathomechanisms and joint motion quality. Due to the prevalence of particular disturbances, our analysis is focused on LPCS, CMP and OA.

## Methods

### Study population

Seventy-three patients (24 males and 49 females) with PFJ disorders (21 with LPCS, 30 with CMP and 22 with OA) were enrolled in the study. All subjects were experiencing anterior knee pain and therefore underwent routine diagnosis by medical interviews, physical examination and imaging (X-rays or MRI), which was interpreted by an expert radiologist. Briefly, patients with LPCS were identified on the basic of comprehensive physical examination including patellar mediolateral glide/mobility, Q-angle, iliotibial band tightness, vastus medialis obliquus weakness and tangential X-ray[21, 22]. Patients with stages II and III of CMP were classified according to criteria of the International Cartilage Repair Society[23] by MRI imaging. OA patients were selected on the basis of clinical/radiological data and the fulfillment of the American College of Rheumatology Subcommittee derived criteria[24]. All patients were recruited from the inpatient and outpatient populations of the Opole Voivodship Medical Centre and Opole Voivodship Hospital, Poland.

Thirty-two unrelated healthy volunteers (10 men and 22 women) possessing neither knee disorders nor pain (analyzed in physical examination, but without radiological exclusion of the cartilage pathologies) served as a control group. Acute inflammation of the knee joint as well as a history of meniscal tear, knee ligament/tendon ruptures, muscle injuries and traumas excluded individuals from the study.

For detailed characteristic of subjects see Table 1. The assessment groups were matched except for OA patient group, which had higher mean age, weight and BMI than patients from other evaluated groups.

**Table 1:** **Characteristic of assessment groups**

	N	Males/Females	Age (years ± s.d.)	Height (cm ± s.d.)	Weight (kg ± s.d.)	BMI
Controls	32	10/22	35.6 ± 5.6	168.4 ± 7.8	69.9 ± 12.1	24.6 ± 3.4
Patients						
With chondromalacia	30	7/23	38.9 ± 7.8	167.8 ± 7.8	71.8 ± 14.6	25.5 ± 4.5
With lateral compression syndrome	21	8/13	34.5 ± 7.2	167.4 ± 8.3	72.9 ± 13.5	25.9 ± 3.4
With osteoarthritis	22	9/13	56.8 ± 7.9	168.5 ± 9.9	83.8 ± 12.8	29.5 ± 3.9

*Abbreviations:* *N* number of cases, *BMI* body mass index, *s.d.* standard deviation.

The project was approved by the Ethics Committee of Opole Voivodship. Signed informed consent was obtained from all tested persons.

### Vibroacoustic signal assessment

**Figure 1:**
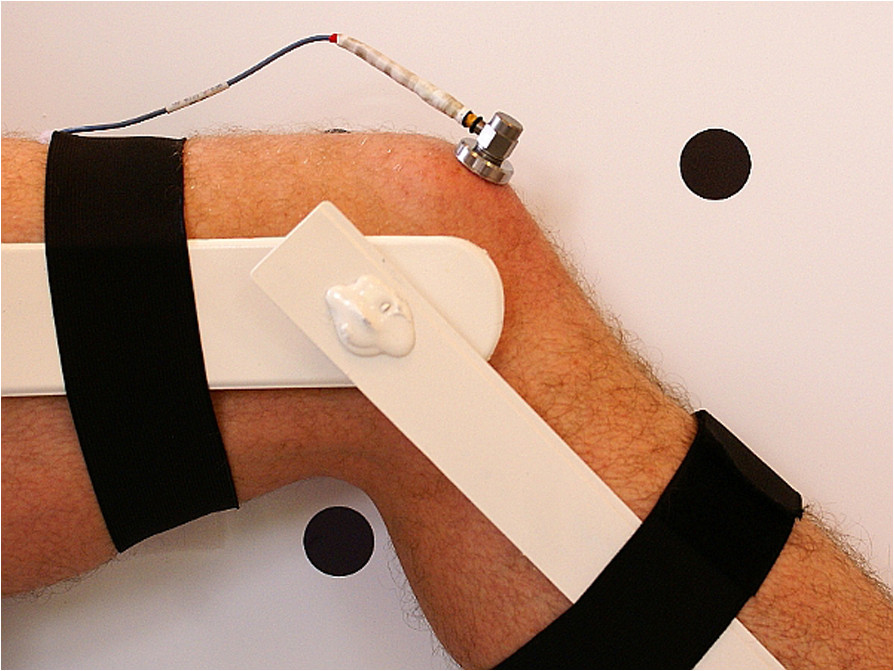
**Correct attachment of vibroacoustic sensor.**

The vibroacoustic signals generated during flexion/extension motion of PFJ were collected by acceleration sensor model 4513B-002 with a multi-channel Nexus conditioning amplifier (Brüel & Kjær Sound & Vibration Measurement A/S, Denmark). The periodicity of recorded signals was between 0.7 Hz and 1000 Hz with a 10 kHz sampling rate, and a 50 Hz threshold for high-pass filtration of obtained signal was used.For each knee, assessment of the VAG signal was performed with a sensor placed, in a seated position, 1 cm above the apex of the patella (Figure 1), and the following procedure: (i) loose hanging legs with knees flexed at 90°; (ii) full knee extension from 90° to 0°; (iii) re-flexion (from 0° to 90°) in sitting position, and a 6-second period. Both flexion/extension motion and measuring condition constant velocities were kept at 82 beats per minute with a metronome.

Parameters related to features of signal, including variability and frequency, were calculated using MATLAB software (The MathWorks, MA, USA). The variability of the VAG signal was assessed by computing the mean-squared values of the obtained signal in fixed-duration segments of 5 ms each, and then by computing the variance over the entire duration of the signal (VMS)[20, 25]:

$$VMS=\frac{\sum_{i=1}^{n}{\left(MS_{i}-\bar{MS}\right)}^{2}}{n-1}$$

where:$MS=\frac{\sum_{j=1}^{k}X_{j}^{2}}{k}$; *n* – number of samples in the VAG signal; *k* = 50 samples (5 ms) – fixed-duration non-overlapping segments of a given VAG signal; *MS*_*i*_ – consecutive mean-squared values in every segment;$\bar{MS}$ – mean of MS_i_; *X*_*j*_ – value of a signal in a segment.

For signal amplitude analysis the parameter R4 was used. Because there were 4 full flexion/extension motion cycles, R4 was calculated as the difference between the mean of four maximum values and the mean of four minimum VAG signal values[17]:

$$R4=\frac{1}{4}\sum_{i=1}^{4}r_{max_{i}}-\;\frac{1}{4}\sum_{i=1}^{4}r_{min_{i}}$$

where: r_max_ – maximum signal value; r_min_ – minimum signal value.

The frequency characteristics of the VAG signal were examined by short-time Fourier transform analysis. The short-time spectra were obtained by computing the discrete Fourier transform of segments of 150 samples each, the Hanning window and a 100-sample overlap of each segment. The spectral activity was analyzed by summing the spectral power of the VAG signal in two bands: 50–250 Hz (P1) and 250–450 Hz (P2)[17].

### Statistical analysis

Multiple group comparison was performed using the one-way ANOVA test, and Tukey range analyses for unequal sample size were applied as post-hoc tests when significant interactions were identified.

All analyses were performed using the Statistica software package v. 9.0 (StatSoft, OA, U.S.A), and p values < 0.05 were considered significant.

## Results

The mean values of VAG signal parameters registered from 86 knees with disorders within the PFJ and 64 control knees are shown in Table 2. One-way ANOVA tests indicated significant differences in subject groups means for all analyzed parameters (VMS: *F* = 19.81, p < 0.001; R4: *F* = 98.66, p < 0.001; P1: *F* = 42.77, p < 0.001; P2: *F* = 26.94, p < 0.001). At the second stage of the analysis of variance, the post-hoc analysis showed that signals recorded for patients suffering from PFJ disorders differ from the controls group. Also, differences between particular disturbances of knee joint were observed.

**Table 2:** **Parameters of vibroacoustic signal in patient and healthy control groups**

	VMS	R4	P1	P2
Controls (C), N = 64	0.003 ± 0.007	1.609 ± 1.121	1.56 ± 1.79	0.24 ± 0.39
Patients				
With chondromalacia (CMP), N = 35	0.178 ± 0.311	5.422 ± 3.021	23.89 ± 22.57	6.49 ± 8.99
With lateral compression syndrome (LPCS), N = 25	0.423 ± 0.413	7.855 ± 2.693	25.73 ± 17.66	3.13 ± 2.68
With osteoarthritis (OA), N = 26	0.869 ± 1.076	9.106 ± 2.167	60.28 ± 44.34	10.99 ± 7.54
P-value				
CMP vs C	0.454	<0.001	<0.001	<0.001
LPCS vs C	0.015	<0.001	<0.001	0.240
OA vs C	<0.001	<0.001	<0.001	<0.001
CMP vs LPCS	0.306	<0.001	0.991	0.130
LPCS vs OA	0.008	0.170	<0.001	<0.001
CMP vs OA	<0.001	<0.001	<0.001	0.016

Abbreviations: VMS, variance of the mean squares calculated in 5 ms windows; R4, the difference between the mean of four maximum and the mean of four minimum values; P1, P2, power spectral density bands: 50–250 Hz and 250–450 Hz, respectively; N, number of knees.

**Figure 2:**
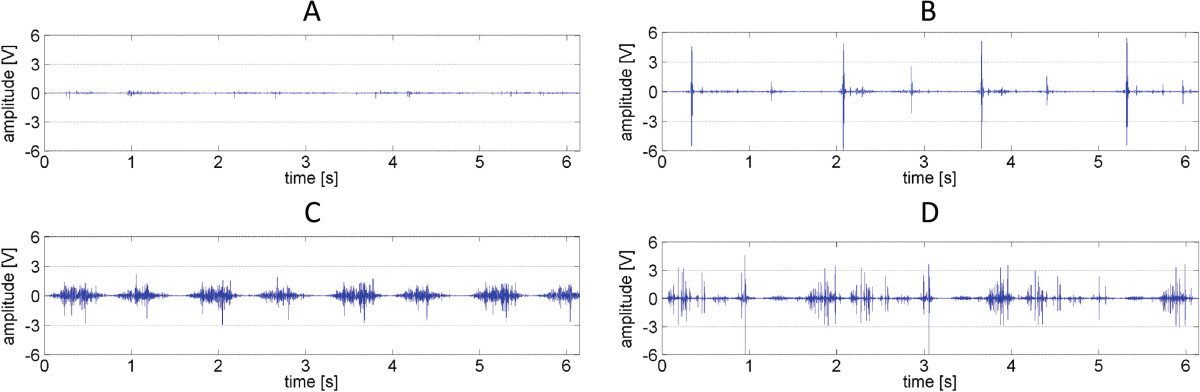
**Time series specific for particular PFJ disorders. (A)**, control healthy knee; **(B)** knee with lateral patellar compression syndrome; **(C)** knee with chondromalacia; **(D)** knee with patellofemoral joint osteoarthritis.

**Figure 3:**
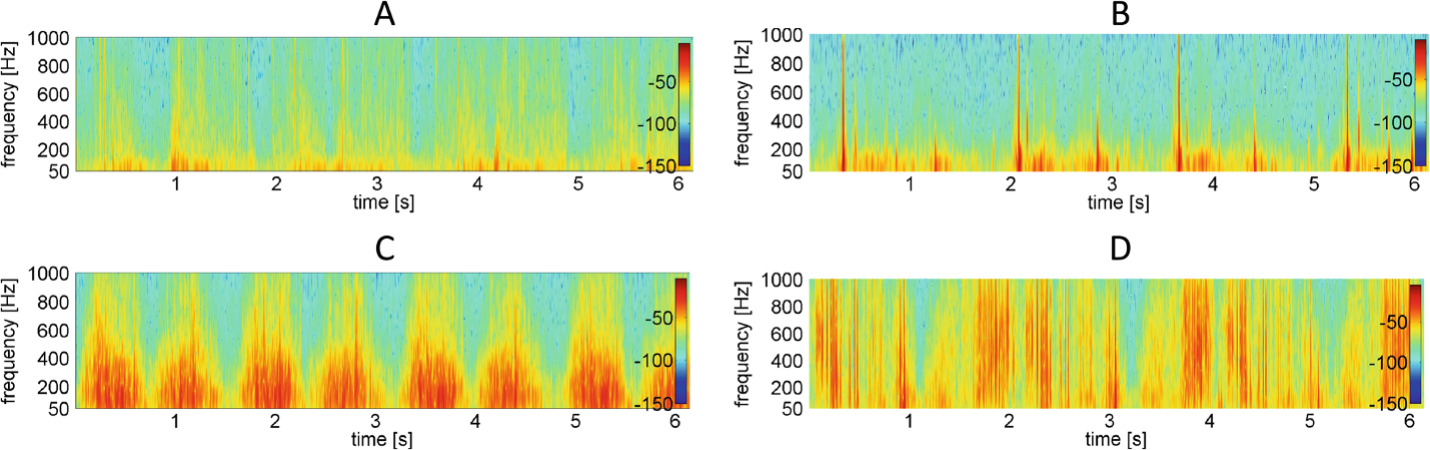
**Signal time-frequency analysis representative for particular PFJ disorders. (A)**, control healthy knee; **(B)** knee with lateral patellar compression syndrome; **(C)** knee with chondromalacia; **(D)** knee with patellofemoral joint osteoarthritis.

Plots of recorded signals typical for healthy control knees are characterized by both small amplitude and low variability, but short single peaks were present (Figures 2A and3A). This feature corresponds with low values of analyzed parameters (Table 2). In comparison to controls, patients with PFJ disorders generated signal with higher amplitude and variability (Figures 2B-D and 3B-D). Therefore signals recorded for CMP, LPCS and OA patient groups were characterized by significantly higher values, especially in R4 and P1 parameters, in comparison to the control group (Table 2).

**Figure 4:**
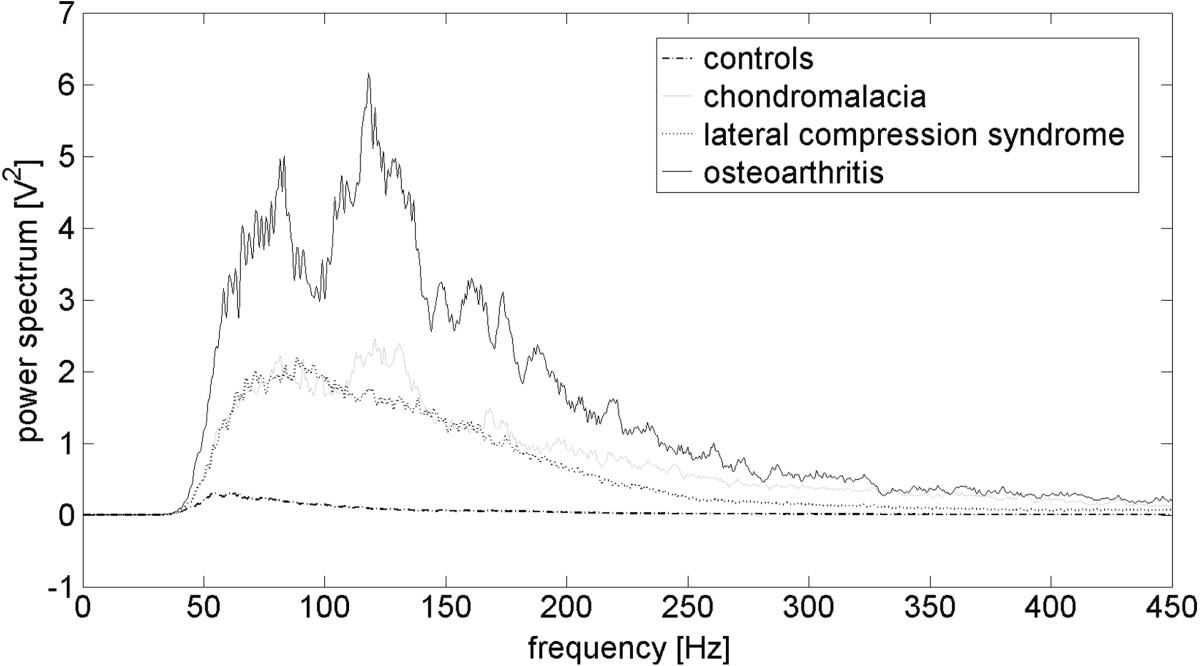
**Power spectrum distribution in VAG signal for each analyzed group.**

Moreover, post-hoc comparisons showed that various types of PFJ disturbances differed from others. It is also illustrated by different patterns of vibroacoustic signal course (Figure 2B-D). Signals recorded for patients with LPCS were characterized by presence of occasional peaks, however, in contrast to healthy knees, these peaks have higher amplitude (Figure 2A and B). Due of this, significantly higher values of VMS, R4 and P1 parameters in comparison to controls were observed. Signals generated by LPCS and CMP knees visually are not similar (Figure 2B and C), however only one differencing factor is parameter R4. Signals typical for the OA group were characterized by even higher variability and the presence of more single peaks (Figure 2D). This phenomenon is confirmed by the highest values of analyzed parameters: OA and CMP groups showed differences in all analyzed parameters. Mean values of R4, P1 and P2 parameters were about twice as low in the CMP group. In addition, VMS, P1 and P2 values were statistically significantly higher in OA than in LPCS (Table 2).Distributions of frequency spectrum for all analyzed groups are shown in Figure 4. Differences in the power spectrum between groups with PFJ disorders (especially OA) and the control group were observed. No difference was found in P1 and P2 values between LPCS and CMP groups; however, in the CMP group a higher VAG signal frequency in the range of 100–150 Hz and above 200 Hz in comparison to LPCS was generated.

## Discussion

The aim of this study was to examine the quality of joint motion related to distinct orthopedic pathologies (LPCS, CMP and OA) of the PFJ in vibroacoustic analysis. Our results confirm that VAG signals generated by abnormal knees differ from healthy control knees[10, 14, 17, 26–28]. It has been postulated that this phenomenon is a result of biomechanical disturbances including cartilage deterioration, leading to impairment of joint motion quality[20, 29]. However, to classify the VAG signals according to articular surfaces conditions the researchers use various modelling techniques[13]. Herein, VAG signal courses were described using four parameters. Variability was illustrated by VMS as described by Rangayyan[20, 25] as well as, due to non-stationary and multi-component nature of VAG signals, the frequency was analyzed. According to Tanaka et al.[28] the frequency analysis was performed for two bands[28], in the range of 50–250 Hz and 250–450 Hz for P1 and P2 parameters, respectively. In addition, the parameter R4, a derivative of range, for signal amplitude analysis was used[17].

Previous research suggests that vibroacoustic signals can distinctly characterize patients with different knee diseases[19, 30]. Reddy et al.[19] found that patients with CMP and OA as well as rheumatoid arthritis generate specific VAG signals. In our study, we analyzed three types of PFJ disorders, and confirmed this phenomenon for CMP and OA in larger patient groups. Additionally, we observed abnormal signal course, which is typical for LPCS knees, while Reddy et al. showed VAG signal course associated with rheumatoid arthritis, an inflammatory joint disease[19].

VAG signal patterns characteristic for mentioned disorders seem to be a result of distinct and disturbance-related pathomechanical backgrounds leading to impairment of joint motion quality. The PFJ disorders are usually characterized by anterior knee pain caused by increased subchondral bone stress and/or cartilaginous lesions[21]. In LPCS, the anterior knee pain seems to be a result of patella maltracking leading to increased compression as well as friction between patella and lateral condyle of the femur. Chronic LPCS may lead to deterioration within articular cartilage (chondromalacia), however, in early stages of the disease only limited presence of chondral structure changes is postulated[31]. In our study, LPCS-related VAG signal course is closer to healthy/physiological knees in most of the created time series. Similar pattern was observed by Reddy et al.[19] in rheumatoid arthritis patient group and the authors suggest that such feature may result from not yet eroded articular surfaces (it has been postulated that extensive erosion begins at a later stage as a rheumatoid arthritis progression). In our study, LPCS group was recruited in relatively early stages of the disturbance what is reflected in VAG signal pattern in accordance to rheumatoid arthritis-related finding[19]. LPCS is additionally characterized by grinding sensation which could explain presence of local and brief peaks, which are replicable in each flexion/extension motion cycle. These peaks determine high value of R4 parameter, probably associated with lateral compression of the patella. Interestingly, in comparison to controls, any differences in P2 parameter was found. It could be postulate that P2 higher value is typical for other disturbances associated with cartilage structural changes, including CMP.

CMP is characterized by softening and fibrillation of particular layers of hyaline cartilage[32]. Our analysis was performed in knees with II and III stage of chondromalacia, which means that deeper chondral layers were already exposed. These layers, when compared with the superficial zone, possess distinct structure and function – collagen fibres are loosely packed in oblique and vertical orientations for self-amortization[33]. The exposure of deeper layers could increase the friction coefficient during relative movement of articular surfaces, which is observed in physical examination as vibroacoustic sensations[34, 35]. The occurrence of this phenomenon was confirmed by us in power spectral analysis showing escalated participation of high frequency in signals recorded from knees of patients with CMP. Clinically, it is not known if friction is more prominent than in osteoarthritis, however, Reddy et al.[19] reported that CMP generates the most prominent vibrations. In contrary, in our study among the four analyzed groups this phenomenon is characteristic for OA.

It may be considered that early LPCS and CMP are functional and structural disorders, respectively. Whereas, osteoarthritis seems to impose both of the mentioned features, because soft tissues’ tightness leads to an increased load on articular surfaces, which provides to subchondral stress. This is associated with degenerative changes of chondral structures and declining lubrication of articular surfaces leading to limited possibilities of reducing friction[36]. As a result, joint motion generates a VAG signal with high amplitude and frequency. Thus, the dual character of OA (functional and structural abnormalities) creates signals with composed attributes (high frequency as well as high amplitude) typical of LPCS and CMP patterns of the VAG signal. However, the mean age of OA patients and other analyzed groups significantly differed (56.8 vs about 35 years), which could have had an impact on the VAG signal pattern. In the OA group impairment of joint motion quality may be discussed as a result of pathological cartilage degeneration as well as the process of physiological senescence.

In summary, the VAG method seems to reflect qualitative features of articular surface movements and vibroarthrographic signal analysis may objectively expand knowledge on joint motion quality in particular PFJ disorders.

## Conclusions

We have shown that particular disorders of the PFJ are characterized by a typical vibroacoustic pattern of waveforms. However, further work is needed to determine whether the sensitivity and specificity of this method are sufficient for clinical application, as a tool for analysis of different kinds of crepitus which are the result of impairment of joint motion quality.

### Consent

The person in the Figure 1 has specifically provided consent to publish their image, the figure was created for the purposes of this study.

## Authors’ original submitted files for images

Below are the links to the authors’ original submitted files for images. Authors’ original file for figure 1 Authors’ original file for figure 2 Authors’ original file for figure 3 Authors’ original file for figure 4

## Abbreviations

PFJ: Patellofemoral joint
OA: Osteoarthritis
CMP: Patellar chondromalacia
LPCS: Lateral patellar compression syndrome
VAG: Vibroarthrography
MRI: Magnetic resonance imaging
BMI: Body mass index
VMS: Variance mean square.
