# Supplementary figures and images for: Joint motion quality in vibroacoustic signal analysis for patients with patellofemoral joint disorders
Source: BMC Musculoskelet Disord. 2014 Dec 12;15:426. doi: 10.1186/1471-2474-15-426 (PMC4295352; doi:10.1186/1471-2474-15-426)

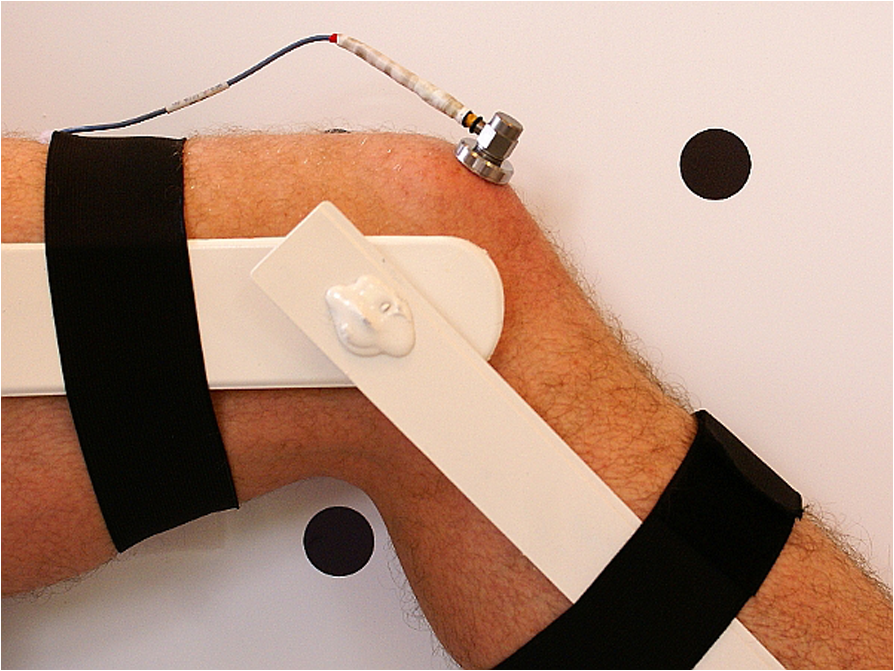

Supplement: Supplementary file 1 — Authors’ original file for figure 1 [file 12891_2014_2355_MOESM1_ESM.tif]

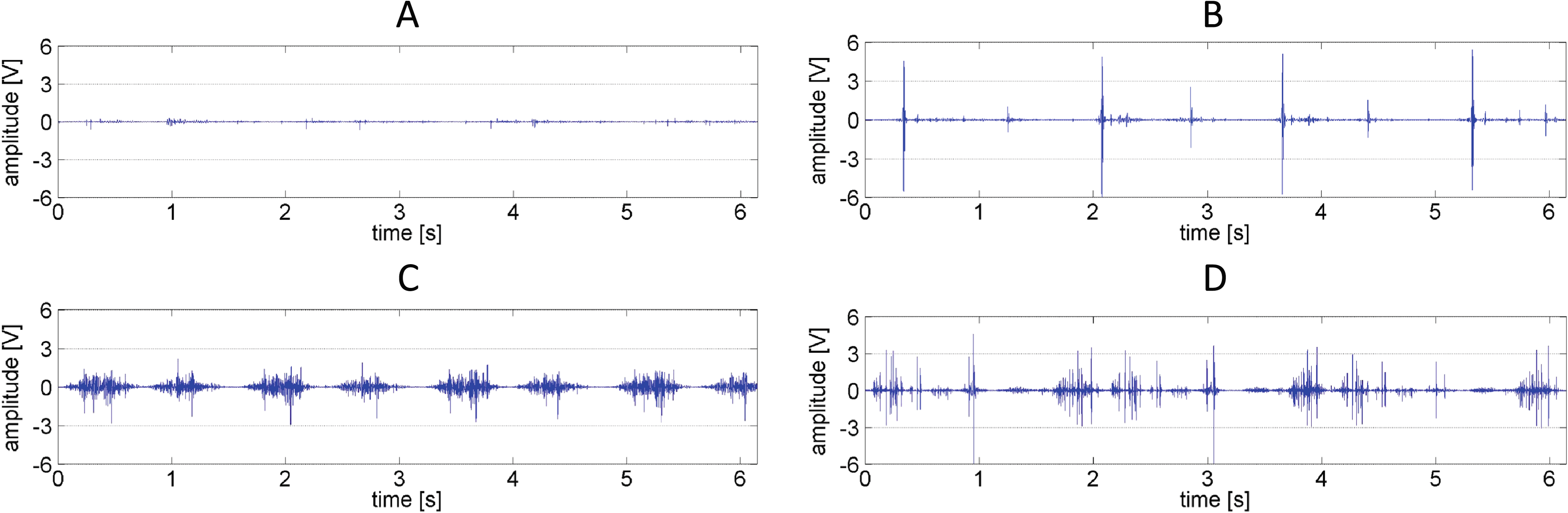

Supplement: Supplementary file 2 — Authors’ original file for figure 2 [file 12891_2014_2355_MOESM2_ESM.tif]

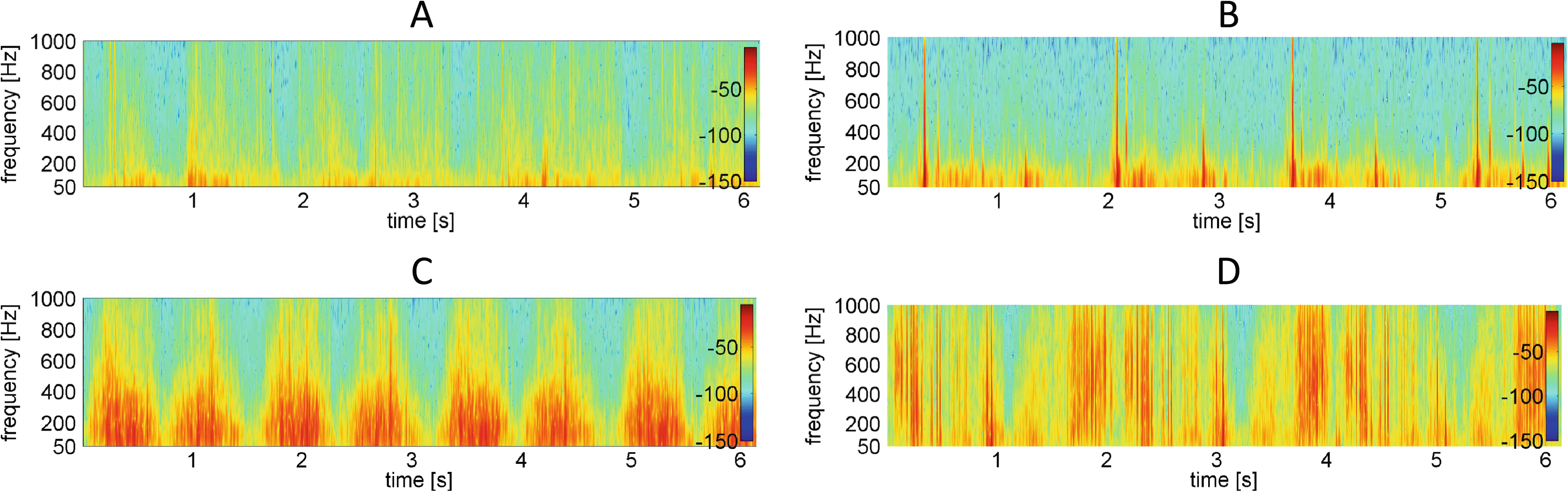

Supplement: Supplementary file 3 — Authors’ original file for figure 3 [file 12891_2014_2355_MOESM3_ESM.tif]

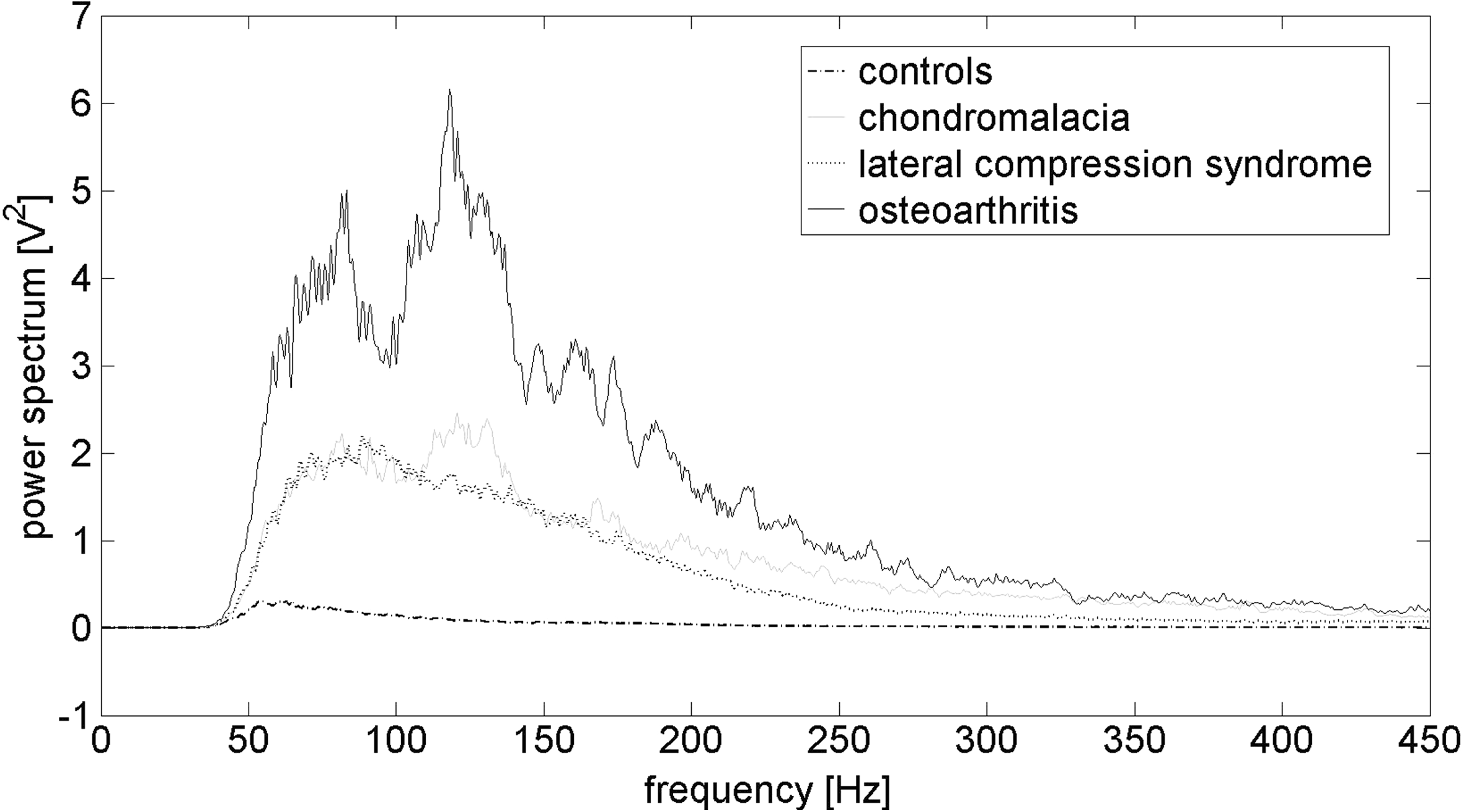

Supplement: Supplementary file 4 — Authors’ original file for figure 4 [file 12891_2014_2355_MOESM4_ESM.tif]
